# Phytochemicals as Biopesticides against the Pinewood Nematode *Bursaphelenchus xylophilus*: A Review on Essential Oils and Their Volatiles

**DOI:** 10.3390/plants10122614

**Published:** 2021-11-28

**Authors:** Jorge M. S. Faria, Pedro Barbosa, Paulo Vieira, Cláudia S. L. Vicente, Ana Cristina Figueiredo, Manuel Mota

**Affiliations:** 1 INIAV, I.P., National Institute for Agrarian and Veterinarian Research, Quinta do Marquês, 2780-159 Oeiras, Portugal; cvicente@uevora.pt; 2 NemaLab-MED, Mediterranean Institute for Agriculture, Environment and Development, Institute for Advanced Studies and Research, Évora University, Pólo da Mitra, Ap. 94, 7006-554 Évora, Portugal; pedronematology@gmail.com (P.B.); mmota@uevora.pt (M.M.); 3 School of Plant and Environmental Science, Virginia Tech, Blacksburg, VA 24061, USA; pvieira@vt.edu; 4 Centro de Estudos do Ambiente e do Mar (CESAM Lisboa), Centro de Biotecnologia Vegetal (CBV), Faculdade de Ciências da Universidade de Lisboa, DBV, C2, Piso 1, Campo Grande, 1749-016 Lisboa, Portugal; acsf@fc.ul.pt; 5 Departamento de Biologia, Escola de Ciências e Tecnologia, Universidade de Évora, 7002-554 Évora, Portugal

**Keywords:** bioassays, biopesticides, phytochemicals, pine wilt disease, pinewood nematode, sustainable pest management

## Abstract

The impacts of a rapidly changing environment together with the growth in global trade activities has promoted new plant pest pandemic events in forest ecosystems. The pinewood nematode (PWN), *Bursaphelenchus xylophilus*, causes strong worldwide economic and ecological impacts. Direct control is performed through trunk injection of powerful nematicides, however many of these (hemi)synthetic compounds have raised ecological and human health concerns for affecting non-target species and accumulating in food products. As sustainable alternatives, essential oils (EOs) have shown very promising results. In this work, available literature on the direct activity of EOs against PWN is reviewed, as a contribution to advance the search for safer and greener biopesticides to be used in sustainable PWD pest management strategies. For the first time, important parameters concerning the bioassays performed, the PWNs bioassayed, and the EOs used are summarized and comparatively analyzed. Ultimately, an overview of the chemical composition of the most active EOs allowed to uncover preliminary guidelines for anti-PWN EO efficiency. The analysis of important information on the volatile phytochemicals composing nematicidal EOs provides a solid basis to engineer sustainable biopesticides capable of controlling the PWN under an integrated pest management framework and contributes to improved forest health.

## 1. Introduction

Over the last few decades, forest health management has been increasingly challenged by the combined effects of intense environmental alterations, imposed by climate change, and a growing number of highly infectious pathologies, triggered by viruses, bacteria, fungi, nematodes, and insect herbivores. The continuous expansion of global trade activities has accelerated the spread of pests and pathogens to new ecosystems. New trading routes and increased shipping activities have contributed to the establishment of largely unconstrained passageways for invasive pests, which have contributed to several epidemic events [1]. Plant parasitic nematodes (PPN) are among the most widespread and damaging global pests in agronomy and forestry. An estimated 12% loss in yield can be attributed to the activity of PPNs, which is more than twice that caused by insect pests [2]. The pinewood nematode (PWN), *Bursaphelenchus xylophilus* (Steiner & Buhrer 1934), is classified as one of the top 10 PPNs with the highest global economic and scientific importance [3]. This migratory plant endoparasite has gained increased attention after its recognition as the causal agent of pine wilt disease (PWD), a pathology responsible for the devastation of vast pine stands in Asian countries [4,5,6]. As a forest pathogen, the PWN is autochthonous to North America, where it poses little threat to the native conifer trees. However, in its native range, it can become extremely damaging to non-native pine species. In the beginning of the 20th century, it was introduced to the susceptible pine forests of Japan, possibly transported in imported wood products used in increasing trade activities, and has since caused massive ecological, economic, and cultural impact [5,7]. Despite a great investment in several disease control measures, PWN has spread to China (1982) and Korea (1988) and was detected, in 1999, across the globe, in Portugal at the European Atlantic shores [8]. This prompted the European and national authorities to swiftly initiate a phytosanitary strategy with the purpose of controlling and eradicating the PWN at its introduction site [9]. Forest conservation authorities implemented the National Eradication Programme for the Pinewood Nematode (PROLUNP) with the primary objective of limiting PWD dispersion through the surveillance of national wood transportation, regulating wood products export, eliminating symptomatic trees, establishing buffer zones, and controlling insect vector populations. Unfortunately, containment efforts were unsuccessful and the PWN was detected in Madeira island and Spain in the following years [10,11,12]. The complete continental area of Portugal is now quarantined, and Spanish border areas are on alert, conducting regular surveys in bordering forests.

Currently, the direct application of (hemi)synthetic pesticides through trunk injection is believed to be one of the most powerful direct PWD control strategies and is amply used in Asian countries [13]. However, most pesticides can be harmful to non-target organisms and have been consecutively withdrawn, due to serious environmental and human health concerns [14]. Strong pressures on the development of improved ecological biopesticides has prompted researchers to explore environmentally friendlier natural compounds with increased anti-nematode properties which are, at the same time, cost-effective [15,16]. Preliminary breakthroughs have been achieved by screening highly active plant natural compounds that show direct activity against the PWN. Essential oils (EOs) stand out for being complex mixtures of natural compounds that have the advantage of being highly active, while not accumulating in the environment and having a broad range of activities, which diminishes the risk of developing resistant pathogenic strains [17]. Research on nematicidal EOs has been mainly performed in the most affected countries in Asia and Europe. EOs have been screened with remarkable success against PWN, sometimes reaching higher activities than commonly-used synthetic chemical nematicides [18]. Nevertheless, information on successful nematicidal EOs is mostly scattered, and the methodologies used can be diverse, which turns comparing and drawing conclusions into a difficult task. Thus, it is important to have a wide-ranging overview of the parameters that characterize the bioassays employed to analyze significant EOs, the variability of the EOs used, as well as their application and anti-nematode activity against *B. xylophilus*, for potential use in the research of sustainable pest management strategies. In the present work, a comprehensive bibliographic review was performed on the direct activity of EOs on *B. xylophilus*. A thorough survey allowed summarizing available information on the (1) bioassays performed, namely, (a) concentration of solubilizing agents, (b) volume of assay solution, (c) number of PWNs per bioassay, (d) EO concentration applied, and (e) duration of PWN contact with the EO; (2) PWNs bioassayed, namely, (a) origin of pathogen isolate and (b) PWN life stage; and (3) EO sources, namely, (a) family and species of the source plant, (b) plant part used for extraction, (c) plant or EO geographical origin, and (d) EO extraction procedure. Additionally, the main composition of EOs was analyzed in relation to PWN mortality and/or the half maximal effective concentrations (EC_50_) reported. According to the compiled parameters, EOs were hierarchized to pinpoint the most toxic EOs and the potential highest nematicidal EO compounds. Tested EO compounds were additionally summarized and discussed in the scope of their chemical properties and nematicidal strengths.

The present review reports, for the first time, the most important parameters used to ascertain EO activity in direct contact bioassays against PWN and discusses the chemical specifiers potentially responsible for PWN nematotoxicity.

## 2. Pine Wilt Disease and the Pinewood Nematode

PWD is an infectious forest disease, generally lethal to susceptible conifer species, caused by the direct activity of PWN, in which symptoms are worsened with the activity of associated and/or opportunistic pathogenic microbiota [19,20,21,22]. Infected susceptible trees display a reduction in oleoresin flux, progressing to a state of shoot desiccation and drooping, due to mechanisms of cavitation and subsequent interruption of sap transport, and chlorosis, as a result of a collapse in photosynthetic functions, culminating in an overall rapid tree decline [23,24]. In affected countries, PWD has significant economic and environmental impact, with vast annual losses in timber (26 million m^3^ of timber since 1945 in Japan alone), increased costs in disease control and management procedures, as well as irreversible changes to the native forest ecosystems, namely, loss in biodiversity, destruction of wildlife habitats, interference in the conservation soil and water, and conversion of forest ecosystem species [5,6,7,19].

The complex infection mechanism of PWD involves the host pine tree, an insect vector, a parasitic PWN, and associated microbiota. The PWN life cycle can progress through the reproductive or dispersal phases, and displays different feeding habits, phytophagous, and mycophagous, which is characteristic of this species [25,26]. In its mycophagous phase, the PWN feeds on fungi growing on dead or decaying pine wood (usually *Botrytis cinerea*, *Ceratocystis* spp. and *Ophiostoma minus*), rapidly multiplying and completing its life cycle [27,28]. While developing inside the egg, the PWN molts into the first juvenile stage, J1, exiting in its second stage juvenile form, J2. These can only move small distances in search for its fungal food source. As they avidly feed and develop to the third and fourth stages, J3 and J4, respectively, storage reserves are accumulated in the form of neutral lipids [29]. The adult forms soon follow and the cycle repeats.

The decaying pine wood can also be used as a nursery site for various Cerambycid beetle species, that can become colonized after the PWN lodges on the tracheal system of the developing juveniles. The *Monochamus* genus, longhorn beetles, is particularly attractive to the PWN, and infected beetles can disperse the phytoparasite across long distances [26,30]. In the presence of longhorn beetles, J2 molt into their dispersive form. The development of the dispersive pre-dauer, JIII, and dauer stages, JIV, are tightly synchronized with the development of the juvenile vector beetle. The dauer juvenile undergoes morphologic and metabolic changes, relying solely on accumulated reserves. In this state, PWN is exceptionally resilient and survives environmental extremes for long periods [31]. As the juvenile vector emerges from the tree, JIV colonize it in great numbers. Inside its insect vector, PWN can reach new hosts and feeding areas. During the beetle’s maturation feeding, PWN can enter healthy pines through wounds made by the beetle on young tree branches. The exit of juvenile nematodes from the host beetle, and subsequent infection of young pine shoots, is regulated by both its nutritional status and specific chemical cues emitted by the beetle host and/or the susceptible pine tree. In fact, low levels of neutral lipids in the juvenile PWNs were found to be determinant for its attraction to β-myrcene, a pine volatile monoterpene, while higher levels increased its attraction to toluene, a beetle cuticular hydrocarbon [29]. In the new host tree, PWN begins invading resin canals, attacking epithelial cells, and causing great damage while moving through the canal system and rapidly reproducing. Pine wilting can be observed as early as 3 weeks after infection, as a result of reduced oleoresin accumulation and damage to xylem tracheids, promoting embolism throughout the xylem column [23,32]. The tree may collapse within 40 to 60 days after infection and, at that point, can contain millions of nematodes throughout the trunk, branches, and roots. The decaying pine becomes attractive to adult *Monochamus* beetles and, consequently, a source for new infections [30,33].

The intricate characteristics of PWN parasitism creates an overwhelming challenge for the development of successful pest management practices. Due to the ability to complete its entire life cycle within the tissues of a susceptible tree, PWN control is very difficult to achieve and usually mobilizes expensive pest management techniques that are often ineffective.

## 3. Pest Management

Several pest management techniques are currently used against PWD, however no single management strategy can be considered effective in controlling PWN spread. There has been considerable investment in the exploitation of resistant pine species, either for reforestation or in crossbreeding programs that create resistant hybrids with economical value. In addition, breeding resistance in species with naturally variable susceptibility is being successfully performed [34,35,36]. Nevertheless, control tactics involving pines with reduced susceptibility are believed to only show positive results in the long run, meanwhile the disease continues to spread. The most common control strategies used focus on eradicating infested trees and wood, treating wood before its use for exportation or industrial purposes, and controlling the insect vector population. Several control strategies are used for PWN pest management in each affected country, which are mainly concerned with eliminating various life stages of either the PWN or its insect vector. In areas where PWD is identified, quarantine measures are put into effect and several practices are implemented, namely, the establishment of pine free buffer zones, which reduce the spread of vector insects, a tight control of wood movements, and the elimination of forest debris capable of harboring insect vector eggs or larva. Infected trees are cut down and treated by (a) chipping any wood parts to less than 6 mm chips, effectively eliminating any insect pupal chambers, and (b) burying or (c) burning, ultimately eliminating the insect and/or PWN. Infected wood can also be treated by chemical means, by spraying or fumigating wood pieces with pesticides, or by thermal treatment, above 60 °C to eliminate both the insect and nematode [9,37,38].

Insecticidal pesticides can also be used to prevent beetle spread to new infection sites. Aerial and ground spraying of (hemi)synthetic chemicals is a tactic with relatively good efficiency. The most commonly-used pesticides are the organic phosphorous insecticides fenitrothion or fenthion and the carbamate *N*-acetylcysteine (NAC), which act by inhibiting cholinesterase activity, or the neonicotinoid thiacloprid, which acts through neuron hyperstimulation. Although the use of chemical pesticides is highly effective, some reports of increased mortality in birds and plant species as well as accumulation in food products above regulated concentrations have created distrust in their use [37,39]. Alternative measures for controlling the spread of vector beetle populations involve the use of traps with pheromones, namely monochamol, or attractive tree volatiles, such as α-pinene and ethanol, and even biological control using the beetle’s natural parasites or predatory birds [40,41,42].

Considerable efforts have also been employed on the characterization of PWN genome and transcriptome [43]. These omic approaches may provide clues to identify targets for genetic engineering based PWN control. Important breakthroughs have been achieved with the analysis of genes involved in development, reproduction, parasitism, and drug resistance [44,45,46,47]. In addition, the transcriptomic analysis of the effects of novel pesticides or biocontrol agents for the PWN or its insect vector may reveal new mechanisms of activity with higher nematicidal efficiency [48,49].

Chemical control, through trunk injection of powerful nematicides, remains one of the most effective and reliable containment strategies within integrated management and is amply used in the most affected countries. The preventive treatment of tree species through trunk injection can be a sustainable control strategy since it reduces environmental impacts and avoids spray drift in the application of chemical pesticides. In comparison with foliar spraying, a greater quantity of active substance effectively reaches the target pest. Supplying the active chemical directly to the vascular system enables systemic activity while avoiding the root or cuticle barriers. This strategy is commonly used in more restricted urban areas, e.g., gardens or parks, but can also be used in orchards and forests [50]. Directly killing the PWN at its site of action is performed by applying lethal concentrations of commercial pesticides, e.g., morantel tartrate, levamisole hydrochloride, mesulfenfos, or nemadectin [37]. Unfortunately, commonly-used insecticides and nematicides can show toxicity to beneficial microorganisms, to humans and animals, and can accumulate in the soil and in food plants above the regulated levels. In many countries some have been banned due to the associated negative ecological effects [39,51]. With the ban imposed on hazardous pesticides and the recent fear of drug resistance on the PWN, in recent years, research efforts have shifted to the development of an environmentally safer control of invasive PWN populations through the use of biopesticides [18,39,52,53]. Biopesticides are commonly less toxic to non-target organisms and the environment, reducing the impact on biodiversity. There are three major classes of biopesticides; biochemical pesticides, microbial pesticides, and plant incorporated protectants. Biochemical pesticides are naturally occurring compounds or mixtures that control plant pests by interfering with important behavioral or physiological mechanisms, while synthetic products act by directly killing or inactivating the pest [54,55]. The use of natural compounds as ecological biopesticides has gained much attention, particularly the screening of highly active EOs [56]. Several EOs have been screened with promising results, in some cases showing higher activities than commercial nematicides.

## 4. Research on Anti-Pinewood Nematode Essential Oils

Screening EOs against PPNs is a relatively recent field of research. The first report on the activity of EOs against PWN was published in 2005, while against other PPNs was reported only 20 years earlier, in 1985 [57,58,59,60]. In the past few years, a great number of EOs have been studied as nematicides against PWN. Research was largely performed using in vitro direct contact bioassays, since in vivo screening of nematotoxic EOs can be influenced by environmental conditions (that cause variation in, e.g., the uptake, retention, transformation, and degradation of EO active compounds) [61]. In vitro studies allow the compilation of a large amount of biological and chemical information, useful for uncovering potential sources of anti-PWN EOs and successful nematicidal chemical structures. Understanding the various parameters involved in testing EOs against the PWN is important to determine the contribution of each to overall anti-PWN activity. Previous studies have compiled EOs tested against *B. xylophilus* but lack a deep analysis of parameters concerning PWN, characteristics of the bioassay, EO composition, and most importantly, their reported activities [62].

In this review, the most important information on PWN and on the activity of EOs was gathered and compiled to be used as the basis for carefully selecting EO sources, bioassay conditions, and data analysis in future works. The surveyed information, detailed below, was organized according to parameters (1) that characterized the bioassays, namely, (a) concentration of solubilizing agent, (b) volume of assay solution, (c) number of PWNs per bioassay, (d) EO concentration applied, and (e) duration of PWN contact with EO; (2) related to the PWNs used, namely, (a) origin of PWN isolate and (b) nematode life stage; and (3) describing the plant material and source of the EOs, namely, (a) family and species of the source plant, (b) plant part used for extraction, (c) plant or EO geographical origin, and (d) EO extraction methodology. When available, EO composition was retrieved and compared with indicators of activity, namely, PWN mortality and/or EO half maximal effective concentrations (EC_50_). All data retrieved was compiled per bioassay, focusing on activity against PWN, either mortality or EC_50_ values.

### 4.1. Bibliographic Sources

**Figure 1:**
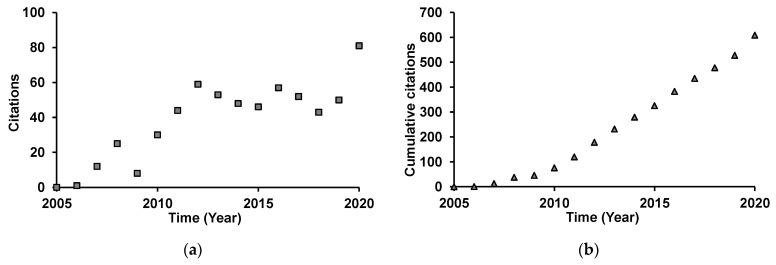
Yearly number of citations (**a**) and cumulative yearly number of citations (**b**) for reports published on the direct activity of essential oils on pinewood nematode.

A thorough bibliographic research was performed with Web of Science search engine [63], in all available databases, on literature reporting on direct contact bioassays, using the topics “*Bursaphelenchus xylophilus*” or “pinewood nematode” and “essential oil”. Thirteen reports were retrieved dating from 2005 to 2013 [18,58,64,65,66,67,68,69,70,71,72,73,74]. The highest number of reports was published in 2007 (5), but since 2013, no additional reports were published on the direct activity of EOs against PWN. Works were mainly published in journals specialized in zoology, agriculture, and biochemistry, and were cited 634 times by a total of 433 publications. From 2005 to 2012, these works were increasingly cited, since then yearly citations stabilized (Figure 1a), probably due to a lack of new publications since 2013. Nevertheless, cumulative yearly citations index is increasing steadily (Figure 1b), which suggests a constant interest in this subject.

### 4.2. Anti-Pinewood Nematode Bioassays

Commonly, direct contact bioassays are performed by subjecting PWN directly to a nematicidal agent. A defined quantity of nematodes is added to a fixed volume of an aqueous solution containing the EO (generally homogenized in a solubilizing agent) and maintained, in controlled conditions, in contact with the nematicidal EO for an allotted amount of time. Most bioassays are performed in multi-well (generally 96) plates that allow performing simultaneous assays. Following, live and dead nematodes are counted, and mortality/toxicity ascertained by mechanically stimulating the immobile nematodes. Lack of movement can be considered a result of toxicity. To determine if immobility is definitive (mortality) or temporary (toxicity), nematodes can be transferred to water to determine if movement can be regained. Although this methodology is fairly simple and fast, it can present several drawbacks, e.g., (a) given the hydrophobic nature of EOs, dilution requires a solubilizing agent, normally non-ionic surfactants or organic solvents, and consequently, its definitive concentration in the assay solution is dependent on the efficacy of the solubilizing agent and the solubilities of the EO compounds [75], (b) the EO compounds possess different volatilities and throughout the experiment may differentially decrease their concentration in the assay solution, (c) lack of PWN movement may not result from nematode mortality and for each toxic EO, restoration of motility may depend on PWN life stage, and (d) counting PWNs under a microscope is still a laborious and straining technique that is easily prone to error and variability, heavily dependent on the observer and their experience. Nevertheless, for a fairly simple and fast determination of EO nematicidal potency, direct contact bioassays remain an excellent starting point.

In the works retrieved, a total of 598 direct-contact bioassays were reported. Overall, the bioassays used to test EO activity on PWN shared common main parameters. Regarding the EO solubilizing agent, the most commonly used was Triton X-100, either alone (54%) or combined with organic solvent ethanol (15%). This non-ionic detergent-type surfactant, known for its capacity to solubilize membrane proteins, increases the penetrating and spreading properties of liquids [70]. Organic solvents were also commonly used and in combination, such as ethanol with castor oil (1%), or alone, namely methanol (21%) or acetone (8%). These polar solvents were employed to increase miscibility of EOs in aqueous solutions. This effect is determined by the chemical nature of the organic solvent but also by the chemical characteristics of the compounds that comprise the EO.

The quantity of nematodes used in each assay was generally dependent on the assay solution volume. When mentioned, the average number of nematodes used per bioassay was 100 (38%), 150 (13%), or 300 (47%), for a final volume of 100 µL (97%). One publication report bioassays performed in 4 mL (3%) but does not specify the number of nematodes [66].

EO concentrations, in the published reports, were either expressed as mg/mL or µL/mL, depending on whether the EO was weighed or its volume measured, respectively. The concentrations tested ranged from 10 to 0.016 (mg or µL)/mL of assay solution. The most frequently used was 2 (mg or µL)/mL (58%), followed by 10 (mg or µL)/mL (16%) and 0.5 (mg or µL)/mL (7%). Commonly, bioassays were performed at the highest EO concentration and later decreased sequentially, until EOs lost activity (decreased mortality), at lower doses.

The time of exposure to the EO varied between 4 h and 72 h, but 24 h was the most used time of exposure (62%), followed by 4 h (31%), 48 h (6%), and 72 h (1%). Given their biodegradable nature, research on fast acting nematicidal EOs should be favored to pinpoint those capable of providing the most adequate biocidal activities.

### 4.3. Bioassayed Pinewood Nematodes

**Figure 2:**
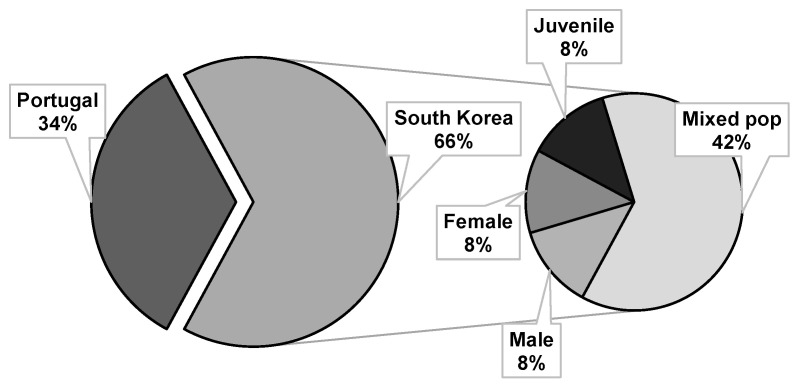
Origin of the pinewood nematode isolates and type of life stage used in direct contact bioassays with essential oils.

The PWN isolates used in the direct contact bioassays were originally either from Portugal (1/3 of bioassays) or South Korea (2/3 of bioassays). The Portuguese isolates were used exclusively in mixed life stage populations while in South Korea, although mixed life stage populations were predominately used (42%), activity on juveniles (8%), females (8%), and males (8%) were also analyzed (Figure 2). Variation in the response of PWN populations, from different geographic origins, to EO nematicidal activity has not yet been explored. Nevertheless, genetic variation has been described for this species. For example, populations from the United States appear to have a higher degree of genetic variation than the ones from Asian countries and Portugal [76,77], which suggests limited instances of colonization. Although Portuguese and Asian isolates appear to be very close and not extremely variable, geographic variation can occur, possibly influenced to a higher degree by anthropogenic factors rather than by natural dispersion through vector insects [78,79,80]. Future research would benefit from addressing the influence of PWN variability on the resulting EO nematicidal activity.

### 4.4. Essential Oils

EOs are commonly termed the essence of a plant. They are most often obtained from aromatic plants that generally grow in tropical and subtropical regions. The fragrant mixtures produced are predominantly comprised of secondary metabolites, whose functions in plants are still debated, but that are often associated to the mediation of the surrounding environment plant-insect, plant-microorganism, or plant-plant interactions [81]. EOs are obtained in the form of a concentrated hydrophobic liquid, at room temperature, slightly soluble in water, and highly soluble in organic solvents. They can be comprised of compounds from a vast range of chemical classes, mainly mono-, sesquiterpenes, and a few diterpenes, phenolic compounds, such as phenylpropanoids, and other groups of compounds [82]. The composition of EOs can be highly dependent on the plant genotype and plant part used, but also on environmental and edaphic conditions, such that plants of the same species in close proximity can produce EOs with different compositions [17]. To be considered an EO, the “product must be obtained from natural raw material of plant origin, by steam distillation, by mechanical processes from the epicarp of citrus fruits, or by dry distillation, after separation of the aqueous phase, if any, by physical processes”, as defined by the International Organization for Standardization (ISO) [83].

EOs are most commonly used in food, perfumery, and pharmaceutical industries, but have also been reported as successful biologically active substances, showing good anti-microbial, anti-viral, fungicidal, anti-malarial, insecticidal, insect repellent, herbicidal, antidepressant, anticancer, antimutagenic, hepatoprotective, anti-inflammatory, antioxidant, anticonvulsant, analgesic, antipyretic acaricidal, and nematicidal activities [17,84,85,86,87,88]. In over 20,000 studies reporting on EO biological activity, ca. 25% were performed on antioxidant activity, 12% on antimicrobial activities, and 11% on insecticidal and insect repellent activities [86]. Additionally, EOs can be a good source for environmentally safer biopesticides or for model compounds in the development of easily biodegradable synthesized derivatives, showing low to negligible phytotoxicity as well as safety for humans [89,90]. EOs do not accumulate in the environment and, as complex mixtures, display diverse biological activities that make them desirable biopesticides, being able to regulate not just the targeted pest but also opportunistic species and resistant strains. This is of particular interest in PWN control since the complex disease symptoms are also commonly linked to associated and opportunistic microbiota [91]. Besides being natural and biodegradable, EOs have also less strict regulatory approval mechanisms for their exploration, due to a long history of use [92].

**Figure 3:**
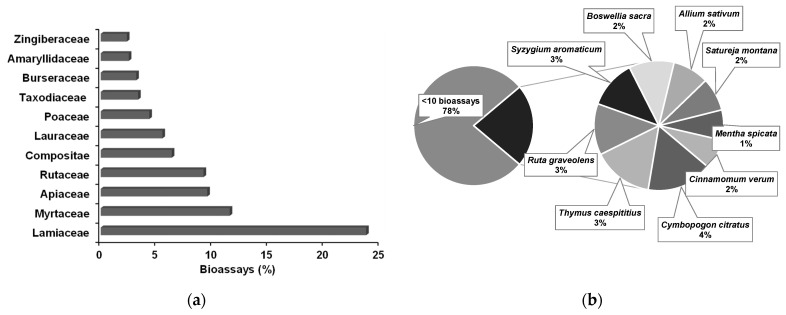
Main families (**a**) and most used species (**b**) of plant sources for essential oils used in direct contact bioassays against pinewood nematode.

In the reports analyzed, a total of 417 EOs were tested in the 598 direct contact bioassays. The EOs were extracted from 217 plant species, belonging to a total of 46 families. Binomial species designations were updated according to the World Flora Online organization [93], that contains a comprehensive listing of species of vascular plants and bryophytes. More than 50% of bioassays used EOs extracted from plants of the Apiaceae, Lamiaceae, Myrtaceae, and Rutaceae families (Figure 3a). From the 217 plant species, those tested less than 10 times ascended to 78%, while the EOs of *Allium sativum* (2%), *Boswellia sacra* (2%), *Cinnamomum verum* (2%), *Cymbopogon citratus* (4%), *Mentha spicata* (1%), *Ruta graveolens* (3%), *Satureja montana* (2%), *Syzygium aromaticum* (3%), and *Thymus caespititius* (3%) were more frequently tested (Figure 3b).

**Figure 4:**
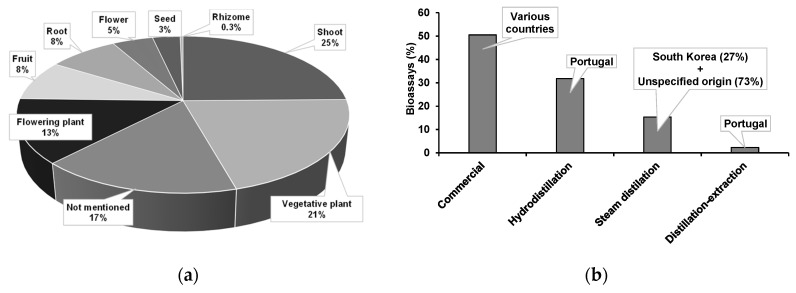
Plant parts (**a**) and countries of origin (**b**) of plants used for extraction (hydrodistillation, steam distillation, or distillation-extraction) of essential oils tested in direct contact bioassays against pinewood nematode.

The plant parts used for EO extraction were either not mentioned (17%) or were shoots or portions of shoots (25%), plants in a vegetative phase (21%), or plants in a flowering phase (13%) (Figure 4a). Other plant parts commonly used were fruits (8%), roots (8%), flowers (5%), seeds (3%), and rhizomes (2 EOs).

More than half of EOs (51%) were acquired from commercial sources and EO extraction methodology was not specified. The remaining were extracted by hydrodistillation (32%), steam distillation (15%), or distillation-extraction (2%) (Figure 4b). It must be noted that extracts obtained by distillation-extraction are not EOs by definition. Nevertheless, they were considered for this analysis since they are valuable sources for mixtures of volatiles. EOs from a commercial origin used plant sources from various countries (namely, Argentina, Austria, Brazil, Bulgaria, Canada, Caribbean, China, Croatia, Egypt, El Salvador, Ecuador, Ethiopia, France, Hungary, India, Indonesia, Iran, Italy, Jamaica, Japan, Morocco, Nepal, New Zealand, the Philippines, Slovenia, Somalia, South Africa, Spain, Turkey, Vietnam, and Zimbabwe). All EOs extracted by hydrodistillation and distillation-extraction were obtained from plants in Portugal, while 27% of those obtained from steam-distillation were extracted from plants of South Korea (in 73% the origin was not specified) (Figure 4b).

Detailing EO origin is very important, nevertheless, the composition of EOs can alter substantially between different species or even geographic origins. Although the main composition of an EO can, most times, be consistent within a species or group, variations can occur depending on a vast number of factors related to the plant used, the selected methodology of extraction, or EO conservation. For example, plant physiological factors, such as the developmental stage, anatomical part, and stress conditions; environmental factors, namely, the season of collection, climate, diseases and pests, edaphic conditions; geographic factors; genetic factors; and EO storage conditions are known to substantially influence EO composition [17]. The occurrence of chemotypes is also a factor of variation in plants of the same species, sometimes being geographically very close [94,95,96].

## 5. Anti-PWN EOs and Their Composition

Generally, the biological activities of EOs are intrinsically linked to the combined effect of their components; to those that show direct biological activity but also to those that have no direct activity on the biological system, but that are capable of influencing resorption, rate of reactions and bioavailability of the active compounds [17,71,97].

**Figure 5:**
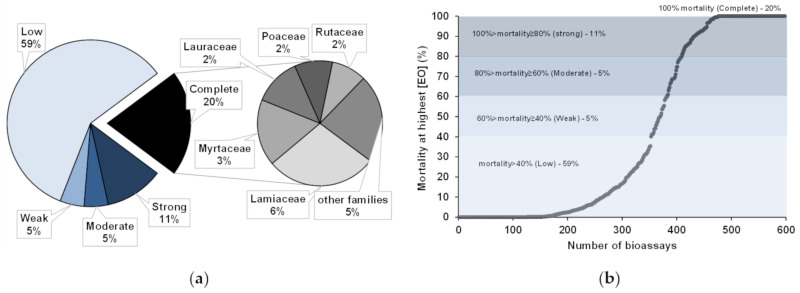
Activities (**a**) and range of pinewood nematode mortality (**b**) reported for essential oils (EOs) used in direct contact bioassays (N = 598). The most frequent families of plant sources for EOs with complete mortality against pinewood nematode are highlighted (**a**).

For the reported EOs, complete activity against PWN, i.e., 100% mortality, was seldomly achieved, and, generally, mortality varied greatly. Activities were defined as (a) complete, at 100% mortality; (b) strong, between 80% and 99% of PWN mortality; (c) moderate, from 60 to 79%; (d) weak, from 40 to 59%; or (e) low, for mortalities under 39% [18]. In most bioassays, low PWN activities were reported (59%), while some showed strong (11%), moderate (5%), or weak (5%) activities (Figure 5a,b). Complete activity (100%) was obtained for 122 bioassays (20%), where EOs from the families Lamiaceae (6%), Myrtaceae (3%), Rutaceae (2%), Lauraceae (2%), and Poaceae (2%) were mainly employed (Figure 5a).

The activity of an EO, under defined conditions, can be expressed by the half maximal effective concentration (EC_50_) parameter, with the advantage of allowing more accurate comparisons with similar studies. Generally, corrected mortality values obtained for various EO concentrations can be fitted to a dose-response curve. Curve fitting allows the determination of various parameters, including the EC_50_, which is the EO concentration that induces a response halfway between the lower and upper limits of the fitted curve. In the works analyzed, nine reports detail EC_50_ parameters. From these, six reports, from South Korea, determined EC_50_ values through Probit analysis [18,65,67,68,72,73], two reports from Portugal used the Weibull function [69,70], and one used a dose–response log–logistic equation [71].

EC_50_ values were reported for the EOs of 28 plant species (Table 1). The most active EO was obtained from *Allium cepa*, the common onion, that showed values as low as 0.012 mg/mL for PWN juveniles and slightly higher values for females (0.014 mg/mL) and males (0.018 mg/mL) (Table 1). Against a mixed life stage population of PWNs, the EOs of *Cinnamomum* species (*C. zeylanicum*, *C. cassia*, and *C. verum*), *Coriandrum sativum*, and *Ruta graveolens* also showed considerable activities.

**Figure 6:**
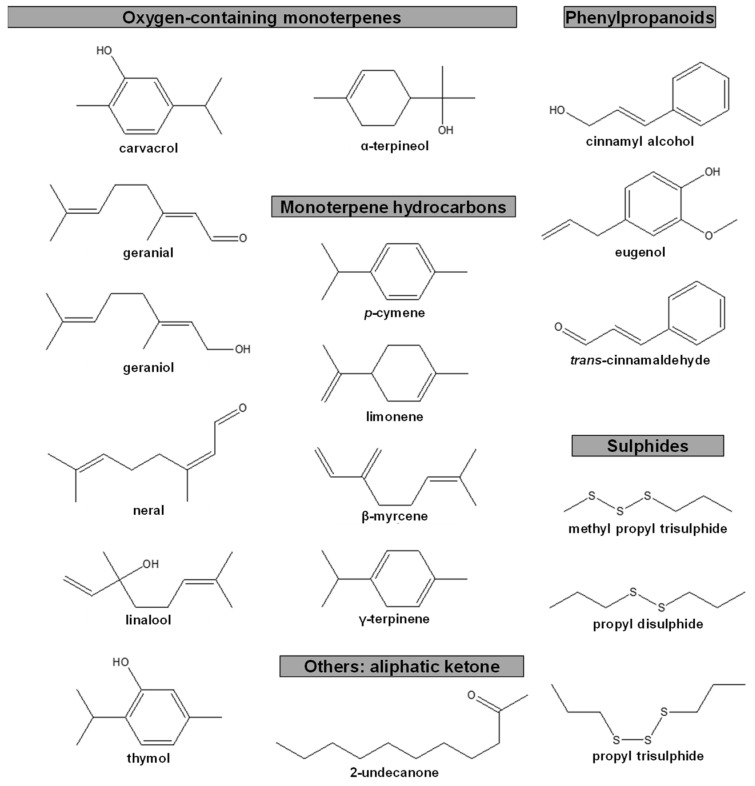
Chemical structure of compounds with percentages ≥15% in essential oils with reported half maximal effective concentrations (EC_50_) against pinewood nematode.

The detailed composition of the EOs used in anti-PWN biological assays was reported in 10 out of the 13 publications identified, however this was largely performed for the most active EOs and in some cases, only for the main compounds [58,64,67,68,69,70,71,72,73,74]. The most active EOs showed compositions rich in compounds with oxygen (O) (monoterpenoids, phenylpropanoids, and others) or with sulphur (S) (sulphides), but also in hydrocarbons (mono- and sesquiterpenes). Some EOs were mainly comprised of one compound (≥75%), e.g., the EOs of *Cinnamomum cassia* (*trans*-cinnamaldehyde), *Cinnamomum verum* (*trans*-cinnamaldehyde), *Cinnamomum zeylanicum* (*trans*-cinnamaldehyde), *Pimenta dioica* (eugenol), *Ruta graveolens* (2-undecanone), *Syzygium aromaticum* (eugenol), *Thymbra capitata* (carvacrol), and *Valeriana jatamansi* (cis-asarone) [58,65,67,69,70,71,74]. The remaining EOs were composed (≥1%) of sulphides or oxygen-containing compounds and combinations of oxygen-containing compounds and hydrocarbons. In those reports where EC_50_ values were detailed, EOs were rich in the aliphatic ketone 2-undecanone; the hydrocarbon monoterpenes *p*-cymene, limonene, β-myrcene, and γ-terpinene; the oxygen-containing monoterpenes carvacrol, geranial, geraniol, linalool, neral, thymol, and α-terpineol; the phenylpropanoids cinnamyl alcohol, eugenol, and *trans*-cinnamaldehyde; or the sulphides methyl propyl trisulphide, propyl disulphide, and propyl trisulphide (see Table 1, Figure 6).

The biological activity of EOs is commonly dependent on the complex mixture of volatiles that compose them. Each EO volatile component can display specific cellular and subcellular activities that can, in the complex mixture that is an EO, be additive, when the biological activity is the sum of each compound activity; synergistic, when the overall EO biological activity is enhanced and is greater than the sum of each compound activity; or antagonistic, when some compounds negatively interfere with the activity of others, leading to a decreased overall EO biological activity. The intensity of these compound relationships in the EO is inherently linked to each component’s concentration and specific activity [97]. Although generally overlooked in most reports, EO compound relationships were preliminary studied by Faria et al. [71]. In this work, the most active EOs were fractionated into two groups through column chromatography, one comprising the oxygen-containing compounds and another with the hydrocarbon molecules, and tested separately. The authors were able to ascertain that the oxygen-containing compounds fraction was responsible for the highest activities, however, depending on the EO, these either resulted in similar (e.g., for *Satureja montana*), higher (e.g., for *Cymbopogon citratus* and *Thymbra capitata*), or lower (e.g., for *Origanum vulgare* and *Thymus caespititius*) EC_50_ values than the respective original EOs, which suggests a synergistic or antagonistic interaction with the hydrocarbon molecules fraction.

### Anti-Pinewood Nematode Essential Oil Compounds

In seven reports, commercially acquired standards, of some compounds that comprised the most active EOs, were tested solely to ascertain the main contributors for EO activity [58,64,67,68,72,73,74]. The EOs with the highest activities were commonly composed of chemicals with very electronegative elements, such as oxygen (O) or sulphur (S) (see Table 2 and Table 3).

The EO components with the lowest EC_50_ values (≤1 mg/mL) were mainly sulphides (diallyl disulphide, diallyl trisulphide, methyl propyl trisulphide, and propyl sulphide), aldehydes (geranial, neral, and *trans*-cinnamaldehyde), and ethers (eugenol, isoeugenol, methyl eugenol, and methyl isoeugenol) (see Table 2). In publications where only mortality percentages were presented, alcohols (decanol, *trans*-2-decen-1-ol, and *trans*-cinnamyl alcohol) also showed high mortalities at low concentrations (Table 3).

Research on the activity of EO components has allowed a deeper understanding of the chemical guidelines that govern the nematicidal strength of certain chemical structures against PWN. For example, in a study that addressed the anti-PWN activity of 26 monoterpenoids commonly occurring in EOs, compounds with phenol, alcohol, or aldehyde functional groups displayed the highest activities while hydrocarbons or ketones were less effective against PWN [98]. Furthermore, primary alcohols were more active than secondary and tertiary alcohols, which means that the position of the hydroxyl group could be related to nematicidal activity. In this study, carvacrol, citronellol, geraniol, menthol, nerol, thymol, citronellal, and citral (a mixture of the geometric isomers geranial, the *trans*-isomer, and neral, the *cis*-isomer) showed higher activities than the commercial nematicide levamisole hydrochloride. Besides the chemical nature of the functional group, the compound isomerism and position of double bonds also appear to influence anti-PWN activity. For example, in similar studies, geranial showed higher activity than its isomer neral, in the same way as *cis*-asarone showed higher activity than its *trans* isomer [73,74]. The position of the double bond of the propenyl group in isoeugenol and methyl isoeugenol granted them stronger activities than eugenol and methyl eugenol [73]. In another study, the monoterpenes (+)-menthol and (−)-borneol displayed high activities against PWN that were weakened after compound glycosylation [99]. However, the anti-PWN activities of thymol and α-terpineol were surprisingly higher after glycosylation. Given that plants can use this enzymatic reaction in the detoxification of xenobiotics, this knowledge can contribute to devising innovative sustainable strategies against PWN [100]. Phenylpropanoids tested against PWN also showed some preferential chemical structures responsible for higher PWN mortality. Anti-PWN activity was followed for derivatives and related compounds of cinnamaldehyde and cinnamic acid [67]. Activities against PWN were reportedly higher for (a) compounds with an aldehyde functional group rather than their respective acids and alcohols, (b) cinnamaldehyde, cinnamic acid, cinnamyl alcohol, and cinnamonitrile rather than in their corresponding saturated compounds, (c) cinnamaldehyde or cinnamic acid than after the introduction of hydroxy, methoxy, or methyl functional groups, and (d) allyl, ethyl, and methyl cinnamates rather than isopropyl and vinyl cinnamates [67]. The study of the chemical specifiers of anti-PWN mortality can be an additional contributor for uncovering novel molecules with improved activity as well as identifying EOs rich in anti-PWN compounds.

## 6. Future Challenges for Research on Anti-PWN EOs

Research on EOs with activity against PWN has evolved progressively, starting with screening a wide range of plants and plant parts, known for the high biological activity of their EOs, and focusing on the chemical characterization of compounds and/or compound interactions responsible for the highest anti-PWN activities. Currently, laboratory screening methodologies for phytochemicals are being improved with the aid of in vitro culture techniques that allow testing compounds in an infection condition where both the host and the parasitic nematode are present [101,102]. The research focus is also shifting to the chemically-guided bioassay of volatile allelochemicals, aiming at improving anti-PWN activity. Being primarily rooted in the search for anti-PWN EOs, many of the compounds studied commonly occur in the reported EOs [103,104]. Uncovering the toxicological characteristics of volatile allelochemicals by tackling the most important structure-activity relationships paves the way for understanding the specific mechanisms of action directing anti-PWN activity in EOs and their compounds [99,105,106,107,108,109]. This approach has yielded interesting results, for example, in the synthesis of multi-functional compounds, such as the dual acting 1-*n*-undecyl-2-[2-fluorphenyl] methyl-3,4-dihydro-6,7-dimethoxy-isoquinolinium chloride, capable of controlling PWN and acting as a fungicide, which has the advantage of debilitating PWN and limiting its fungal food source [110]. The application of many EO or EO compounds in the field is limited by their hydrophobicity and low stability. Recent research is tackling this constraint by, for example, engineering chitosan-coated nanoemulsions of highly anti-PWN natural compounds, namely, dipropyl trisulphide and methyl propyl trisulphide, improving the long-term storage stability and persistence of anti-PWN activity, while maintaining similar EC_50_ values to its original compounds [111]. Future research in the field of nematicidal biopesticides can highly benefit from organic chemistry expertise in linking chemical structure to biological activity to uncover definitive guidelines for increased anti-PWN activity and their subcellular mechanisms of action in PWN.

## 7. Conclusions

The use of natural compounds for the biological control of important pests is extremely advantageous, since it provides eco-friendly biodegradable alternatives to dangerous synthetic chemicals, whose study leads to the development of derivatives with increased activity or specificity; brings recognition to the diversity of natural resources; and promotes sustainable pest management strategies that stimulate the recovery of ecosystem diversity. Against PWN, a high number of EOs have been screened and several have been proven effective. The summarization of the methodologies used to assess the nematicidal activity of EOs permitted determining the most utilized and successful bioassay conditions, and is presented here in detail as a guideline towards a standardization of direct contact bioassays against the PWN. Linking the composition of EOs to their nematicidal activity allowed for uncovering the compounds with the highest nematicidal potential. From the reported EOs, those rich in sulphides or oxygen-containing compounds showed the lowest effective concentrations and are candidates for in vivo testing, under infection conditions, in order to assess efficiency against PWD. The application of the latest developments on anti-PWN EO research allied to the optimization of current methodologies can provide an innovative strategy to establish sustainable pest management practices against PWD.

## Figures and Tables

**Table 1:** Half maximal effective concentrations (EC_50_) reported for essential oils active against various life stages of the pinewood nematode and respective major compounds in relative proportions (≥1%), when available.

EC_50_ [mg/mL (or µL/mL *)]					
Plant Species/Common Name	Mixed Population	Male	Female	Juvenile	Major Compounds (≥1%)
*Acorus calamus* [18] [Sweet flag/Calamus]	2.850				
*Allium cepa* [68] [Onion]		0.018	0.014	0.012	Propyl trisulphide 47, propyl disulphide 34, methyl propyl trisulphide 15, methyl propyl disulphide 3
*Aniba rosaeodora* [18] [Rosewood]	2.990				
*Boswellia sacra* [65] [Frankincense]		0.290	0.260	0.210	
*Cinnamomum cassia* [67] [Cassia/Chinese cinnamon]	0.084				*trans*-Cinnamaldehyde 80, 2-methoxy cinnamaldehyde 13, cinnamyl acetate 4, α-copaene 2, benzaldehyde 1
*Cinnamomum cassia* [67] [Cassia/Chinese cinnamon]	0.085				*trans*-Cinnamaldehyde 91, 2-methoxy cinnamaldehyde 5, cinnamyl acetate 2, *trans*-cinnamic acid 1
*Cinnamomum verum* [18] [Cinnamon]	0.120				
*Cinnamomum zeylanicum* [67] [Cinnamon]	0.064				*trans*-Cinnamaldehyde 99, benzaldehyde 1
*Cinnamomum zeylanicum* [67] [Cinnamon]	0.097				*trans*-Cinnamaldehyde 82, cinnamyl acetate 10, 2-methoxycinnamaldehyde 6, benzaldehyde 2
*Cinnamomum zeylanicum* [67] [Cinnamon]	0.107				*trans*-Cinnamaldehyde 61, eugenol 13, α-terpineol 10, *p*-cymene 8, linalool 3
*Cinnamomum zeylanicum* [67] [Cinnamon]	0.113				*trans*-Cinnamaldehyde 53, limonene 17, cinnamyl alcohol 16, eugenol 13
*Coriandrum sativum* [18] [Coriander]	0.140				
*Coriandrum sativum* [18] [Coriander]	2.760				
*Cymbopogon citratus* [69] [Lemongrass]	0.350				Geranial 43, neral 29, β-myrcene 25
*Cymbopogon citratus* [71] [Lemongrass]	0.456*				Geranial 34, neral 22, β-myrcene 20, geraniol 18
*Cymbopogon citratus* [18] [Lemongrass]	0.570				
*Cymbopogon nardus* [18] [Citronella grass]	2.110				
*Genista tridentata* [69] [Carqueja #]	1.060				1-Octen-3-ol 9, *n*-nonanal 7, linalool 7, *trans*-anethole 5, dodecanoid acid 5, cis-theaspirane 3, 2-undecanone 2
*Litsea cubeba* [73] [Aromatic litsea]	0.504				Geranial 39, neral 30, limonene 15
*Litsea cubeba* [18] [Aromatic litsea]	3.650				
*Melissa officinalis* [18] [Lemon balm]	4.110				
*Nepeta tenuifolia* [65] [Jing Jie]		0.470	0.490	0.410	
*Origanum vulgare* [71] [Oregano]	0.754*				Carvacrol 14, *cis*-sabinene hydrate 14, γ-terpinene 10
*Origanum vulgare* [71] [Oregano]	0.850*				α-Terpineol 40, linalool 16, thymol 12
*Origanum vulgare* [69] [Oregano]	1.210				Carvacrol 36, carvacrol methyl ether 8, β-caryophyllene 2
*Origanum vulgare* [18] [Oregano]	1.420				
*Paeonia × suffruticosa* [65] [Tree peony]		0.320	0.340	0.260	
*Perilla frutescens* [65] [Beefsteak plant/Perilla]		0.530	0.570	0.410	
*Pimenta dioica* [73] [Allspice]	0.609				Eugenol 86, β-caryophyllene 8, methyl eugenol 4, α-humulene 1
*Pimenta dioica* [18] [Allspice]	1.800				
*Pimenta racemosa* [18] [Bay rum tree]	2.270				
*Rosa x damascena* [18] [Damask rose]	4.470				
*Ruta graveolens* [71] [Rue]	0.184 *				2-Undecanone 93
*Ruta graveolens* [70] [Rue]	0.200				2-Undecanone 93
*Ruta graveolens* [71] [Rue]	0.230 *				2-Undecanone 91
*Ruta graveolens* [70] [Rue]	0.230				2-Undecanone 94
*Ruta graveolens* [71] [Rue]	0.232 *				2-Undecanone 94
*Satureja hortensis* [18] [Summer savory]	1.150				
*Satureja montana* [71] [Winter savory]	0.261 *				Carvacrol 64, γ-terpinene 18
*Satureja montana* [70] [Winter savory]	0.340				γ-Terpinene 41, carvacrol 35, *p*-cymene 8, α-terpinene 4, β-myrcene 3, α-pinene 2, α-thujene 2
*Satureja montana* [70] [Winter savory]	0.350				Carvacrol 40, *p*-cymene 20, thymol 15, γ-terpinene 4, borneol 4, terpinen-4-ol 4
*Satureja montana* [69] [Winter savory]	0.380				Carvacrol 39, γ-terpinene 40, *p*-cymene 7, β-myrcene 3, α-pinene 2
*Syzygium aromaticum* [18] [Clove]	0.880				
*Thymbra capitata* [71] [Conehead thyme]	0.265 *				Carvacrol 68, γ-terpinene 11
*Thymbra capitata* [69] [Conehead thyme]	0.500				Carvacrol 75
*Thymbra capitata* [18] [Conehead thyme]	0.820				
*Thymus caespititius* [69] [Tormentelo #]	0.390				Carvacrol 65, carvacrol acetate 11
*Thymus caespititius* [71] [Tormentelo #]	0.972 *				Carvacrol 54, carvacrol acetate 10
*Thymus vulgaris* [18] [Thyme]	0.820				
*Thymus vulgaris* [72] [Thyme]	1.390				Thymol 58, *p*-cymene 18, γ-terpinene 9, linalool 4, carvacrol 3
*Thymus vulgaris* [72] [Thyme]	1.640				Thymol 48, *p*-cymene 18, linalool 11, γ-terpinene 7, limonene 4, camphor 4, terpinen-4-ol 2, carvacrol 2
*Trachyspermum ammi* [73] [Ajwain]	0.431				Thymol 42, γ-terpinene 28, *p*-cymene 24, β-pinene 1

*-Values reported in µL/mL, #-No vernacular English name.

**Table 2:** Pure compounds tested against the pinewood nematode with reported half maximal effective concentrations (EC_50_) values ≤1 mg/mL.

EO Compounds	EC_50_ (mg/mL)
Diallyl trisulphide	0.003–0.004
Propyl sulphide	0.004–0.005
Methyl propyl trisulphide	0.017–0.023
Cinnamyl acetate	0.033–2.766
Diallyl disulphide	0.037–0.047
*trans*-Cinnamaldehyde	0.057
Geranial	0.120
Isoeugenol	0.200
Methyl isoeugenol	0.210
Geraniol	0.430
Eugenol	0.480–1.212
Methyl eugenol	0.517
Neral	0.525
*trans*-Cinnamic acid	0.750

**Table 3:** Pure compounds tested against the pinewood nematode with reported complete mortality (100%) and respective lowest tested concentration at which complete mortality was observed (mg/mL).

EO Compound	Lowest Concentration (mg/mL)
Methyl *trans*-cinnamate	0.063
Decanol	0.200
*trans*-2-Decenal	0.200
Ethyl *trans*-cinnamate	0.250
Methyl propyl trisulphide	0.250
Propyl sulphide	0.250
*trans*-2-Decen-1-ol	0.400
*cis*-Asarone	0.800
*trans*-Cinnamyl alcohol	0.800
Decanal	1.000
Eugenol	1.000
Geranial	1.000
Isoeugenol	1.000
Methyl isoeugenol	1.000
Benzaldehyde	2.000
Dodecanal	2.000
Nonanal	2.000
Octanal	2.000
Undecanal	2.000

## Data Availability

The raw data supporting the findings of this study are available from the corresponding author (Jorge M. S. Faria) upon reasonable request.
